# Spontaneous clearance of chronic hepatitis C is rare in HIV-infected patients after effective use of combination antiretroviral therapy

**DOI:** 10.1371/journal.pone.0177141

**Published:** 2017-05-04

**Authors:** Mario Frias, Antonio Rivero-Juarez, Francisco Tellez, Monserrat Perez-Perez, Angela Camacho, Isabel Machuca, Sandra Lorenzo-Moncada, Pedro Lopez-Lopez, Antonio Rivero, for the Grupo de Estudio de Hepatitis Virales (HEPAVIR) of the Sociedad Andaluza de Enfermedades Infecciosas (SAEI)

**Affiliations:** 1 Infectious Diseases Unit, Instituto Maimónides de Investigación Biomédica de Córdoba (IMIBIC), Hospital Universitario Reina Sofía de Córdoba, Córdoba, Spain; 2 Infectious Diseases and Microbiology Unit, Hospital La Línea, AGS Campo de Gibraltar, Cádiz, Spain; Harvard Medical School, UNITED STATES

## Abstract

**Objective:**

To evaluate the rate of spontaneous resolution of chronic hepatitis C (CHC) infection in a cohort of HIV-infected patients.

**Methods:**

A retrospective analysis of 509 HIV-infected patients with chronic HCV infection was performed at two reference hospitals in Andalusia. The main variable of the study was spontaneous clearance of CHC, defined as a negative HCV RNA result after at least two previous quantitative measurements of HCV RNA separated by a minimum of 12 months.

**Results:**

Of 509 patients, 3 (0.59%; 95% CI: 0.15%-1.6%) experienced spontaneous clearance of CHC. After combination antiretroviral therapy (cART) initiation, two of three cases experienced an increased CD4^+^ count, coinciding with HCV viral clearance. All patients were IL28B CC carriers, 2 were co-infected with HCV genotype 3 (the HCV genotype of the remaining patient was not available).

**Conclusions:**

Spontaneous clearance of CHC is a rare event in the context of HIV/HCV co-infected patients and may be associated with the effective use of cART and thus HIV suppression.

## Introduction

Hepatitis C virus (HCV) causes an infection that can produce both acute and chronic hepatitis [1]. After acute infection, approximately 20–25% of HCV patients experience spontaneous clearance mediated by an immune response [2]. One of the key components of the immune system forming the first line of defense against HCV is cell-mediated immunity, specifically the T lymphocytes and natural killer (NK) cells. The HCV-specific CD4^+^ and CD8^+^ cells are associated with viral clearance via cytolitic or non-cytolitic mechanisms [3]. The NK cells can inhibit virus replication in infected hepatocytes by direct cytotoxicity and the production of inflammatory cytokines, such as interferon gamma [4, 5]. Impaired anti-HCV activity of T- and NK cells therefore could lead to a higher likelihood of developing chronic HCV infection [6, 7]. This could be the reason why HIV-infected patients have lower rates of HCV viral clearance than HCV-monoinfected subjects [8, 9].

The implementation of combination antiretroviral therapy (cART) has considerably improved the life expectancy of patients living with HIV [10]. As a result of achieving sustained virological suppression with cART, the immune system partly recovers and a certain reversal of both the defective T and NK cells can be observed [11]. Consequently, once the immune system recovers, other infections may resolve spontaneously, even in cases of long-term chronic infections such as chronic hepatitis C (CHC) [12]. This situation is defined as spontaneous clearance of CHC and only a few cases have been documented [13–19], which is why little is known about this rare occurrence. The aim of this study was to evaluate the rate of spontaneous resolution of CHC in a cohort of HIV-infected subjects after effective use of cART.

## Material and methods

### Study population

Five hundred and nine HIV/HCV co-infected patients in follow-up at two reference hospitals in Andalusia (southern Spain) between January 2000 and September 2016 were analyzed retrospectively for the achievement of spontaneous clearance of CHC. Those patients identified with spontaneous clearance of CHC had to meet the following criteria: i) they had never been treated for HCV infection, and ii) initiation of cART was not before the follow-up period.

### Variable collection and definition

The main variable of the study was spontaneous clearance of CHC, defined as a negative HCV RNA measurement, preceded by at least two quantitative HCV RNA tests with a minimum of 12 months between them. Clinical, demographic and analytical variables were collected from patients identified with spontaneous clearance of CHC from diagnosis of HCV infection until the end of the study (September 2016). These variables were: liver fibrosis stage, cART regimen, HIV viral load (copies/mL), CD4^+^ count (cells/mL), HCV viral load (IU/mL), HCV genotype and IL28B genotype. Fibrosis stage was determined by liver biopsy or, after 2007, by transient elastography (FibroScan®, Echosens, Paris). In such cases, a liver stiffness value of ≥14.6 kPa was defined as indicating liver cirrhosis [20].

Plasma HCV RNA loads were measured by quantitative PCR assay (CobasTaqMan, Roche Diagnostic Systems Inc., Pleasanton, CA, USA) with a limit of detection of 15 IU/mL. HCV genotype was determined by hybridization assay (INNO-LiPa HCV, Bayer Corp., Tarrytown, NY, USA). HIV viral load was measured by PCR (CobasTaqMan, Roche Diagnostic Systems Inc., Pleasanton, CA, USA), with limit of detection set at 20 copies/mL.

### Statistical analysis

A descriptive analysis was performed. The number of cases of spontaneous clearance of CHC was expressed as a percentage, using a two-sided 95% CI with respect to the overall population calculated on the basis of the exact binomial distribution. GraphPad Prism version 6 (Mac OS X version; GraphPad Software; San Diego, California, USA) was used to calculate percentages and plot graphs.

### Ethical aspect

The study was designed and performed according to the Helsinki Declaration and was approved by the ethics committee of the Reina Sofia University Hospital, Cordoba, Spain. The study did not require informed consent because the patients were not interviewed directly and the information was collected from completely anonymous pre-existing records, thus ensuring the protection of personal data in accordance with Law 15/1999 of 13 December on Personal Data Protection.

## Results

A total of 509 HIV patients with chronic HCV infection were included in the study. Of these, 3 (0.59%; 95% CI: 0.15%-1.6%) patients experienced spontaneous clearance of CHC. The main characteristics of these patients are shown in Table 1.

**Table 1:** Main characteristics of patients with spontaneous clearance of CHC.

	Case 1	Case 2	Case 3
**Age (years)**	34	25	22
**Sex (gender)**	Male	Female	Male
**cART at HCV clearance**	SQV/rtv + 3TC + AZT	SQV/rtv + FTC + TDF	FTC + TDF + EFV
**Nadir CD4**^**+**^ **(cells/mL)**	267	88	148
**CD4**^**+**^ **at HCV clearance (cells/mL)**	651	127	693
**AIDS-indicator conditions in the past**	No	No	No
**IL28B genotype**	CC	CC	CC
**Risk practice**	IDU	Blood transfusion	Blood transfusion
**HCV Genotype**	NA	3	3

3TC, lamivudine; AIDS, acquired immune deficiency syndrome; cART, combination antiretroviral therapy; AZT, zidovudine; EFV, efavirenz; FTC, emtricitabine; HCV, hepatitis C virus; IDU, injection drug user; rtv, ritonavir; SQV, saquinavir; TDF, tenofovir disoproxil fumarate; NA, not available.

The cases are summarized as follows:

### Case 1 (Fig 1A)

A 34 year-old man, an injection drug user (IDU), was diagnosed with HIV infection in 1993. He received neither clinical follow-up nor cART until August 2004. At this point, the patient was asymptomatic, with a CD4^+^ count of 267 cells/mL, an HIV RNA viral load of 86,500 copies/mL and an HCV RNA viral load of 2,280,000 IU/mL. He initiated cART in August 2004 with a regimen of lopinavir boosted with ritonavir (LPV/rtv) plus lamivudine (3TC) and zidovudine (AZT). In June 2005, with a CD4^+^ count of 447 cells/mL, the patient achieved an undetectable HIV RNA viral load. At that point, his HCV viral load remained detectable with an HCV RNA titer of 4,710 IU/mL. In September 2006, the patient was asymptomatic. His HIV and HCV viral loads were undetectable and were still negative at the last visit in May 2016.

**Fig 1:**
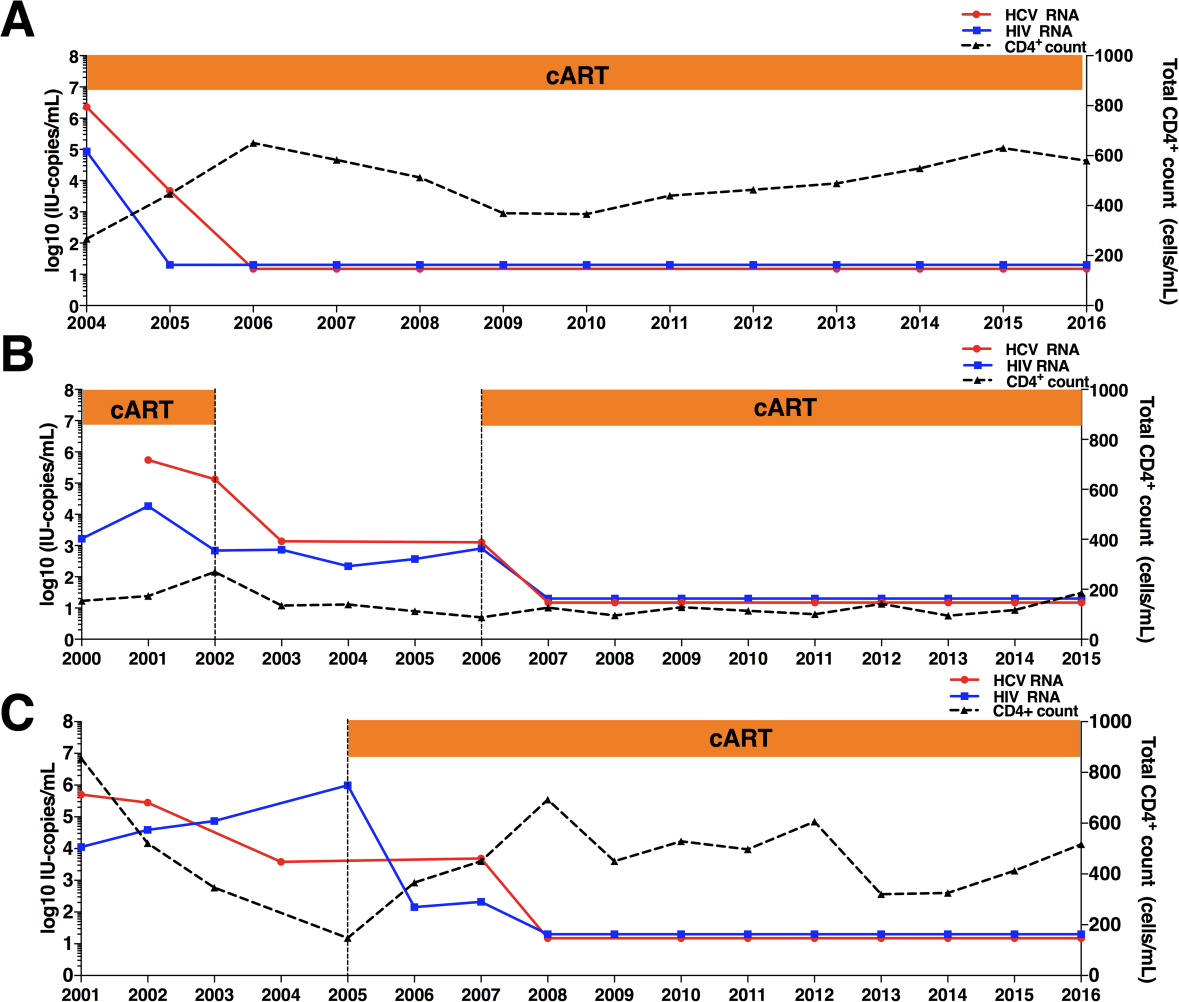
Clinical course of three patients with spontaneous clearance of chronic HCV infection. The figure shows changes in HIV RNA (blue line), CD4+ cell count (dashed black line), and HCV RNA (red line).

### Case 2 (Fig 1B)

A 25 year-old woman was diagnosed with post-transfusion HIV infection in 1987. In April 2000, the patient had a CD4^+^ count of 154 cells/mL with an HIV RNA viral load of 1,610 copies/mL. She initiated cART with efavirenz (EFV) plus AZT and 3TC, but had poor adherence until she voluntarily abandoned cART in February 2002. The patient did not achieve an undetectable HIV viral load during the on-cART period. In August 2001, she was diagnosed with HCV genotype 3 infection, with a viral load of 550,000 IU/mL. Between 2002 and 2006, the patient voluntarily continued interruption of cART and had successive detectable HCV RNA viral loads (between 134,000 and 1,380 IU/mL). In August 2005, a liver biopsy was performed, which was compatible with a diagnosis of cirrhosis. The patient also showed a platelet count of 41,000 cells/mL, total bilirubin of 2.4 mg/dL, albumin of 3.90 g/dL, and an INR of 1.3 (Child-PT score of 6).

Ultrasound findings showed splenomegaly (15cm) and portal hypertension (15mm). In March 2006, the patient had a CD4^+^ count of 88 cells/mL, HIV viral load of 808 copies/mL and HCV with a viral load of 1,270 IU/mL. The patient then initiated treatment with SQV/rtv plus emtricitabine (FTC) and tenofovir (TDF). In April 2007, her CD4^+^ count was 127 cells/mL and she achieved undetectable HIV and HCV viral loads. At that point, the liver stiffness measurement using transient elastography was suggestive of liver cirrhosis (21.8 kPa). Her HIV and HCV viral loads remained negative until May 2015, when the patient died of colorectal cancer.

### Case 3 (Fig 1C)

A 22 year-old man with hemophilia was diagnosed with HIV infection in 1992. In the years that followed, the patient was asymptomatic, his CD4^+^ count was over 800 cells/mL and his HIV viral load was below 10,000 copies/mL without use of cART. In June 2001, the patient showed HCV genotype 3 infection with a viral load of 504,000 IU/mL. At that point, the patient had a CD4^+^ count of 855 cells/mL and his HIV viral load was 11,200 copies/mL. Between 2002 and 2005, the patient showed two consecutive detectable HCV viral loads. In June 2005, the patient experienced a significant drop in his CD4^+^ count (up to 148 cells/mL) and his HIV viral load was 100,000 copies/mL. One month later (July 2005), the patient initiated cART with a regimen of efavirenz (EFV) plus 3TC and didanosine (DDI). In March 2007, cART was changed to EFV plus FTC and TDF showing a CD4^+^ count of 366 cells/mL, HIV RNA viral load of 209 copies/mL and HCV RNA viral load of 4,870 IU/mL. In January 2008, the patient was asymptomatic and both HIV and HCV viral loads were undetectable and remained negative until the last visit in July 2016.

## Discussion

Once chronic HCV is established, the infection is very unlikely to be eradicated without using HCV therapy. However, several cases of spontaneous clearance of CHC have been reported in the setting of HIV/HCV-coinfection, including patients with several years of detectable HCV viral loads [12–19]. In our cohort, we found three cases of chronic spontaneous HCV clearance after effective use of cART. The rate of this rare event was 0.59%, similar to those found in other studies.

The possible mechanisms involved in this event have still not been clarified. Effective treatment with cART suppresses HIV replication and restores the total CD4^+^ count. Immunologic reconstitution as result of successfully implementing cART could therefore be the main factor responsible for the spontaneous clearance of CHC. In our study, *Case 1* (nadir CD4^+^ count, 267 cells/mL) and *Case 3* (nadir CD4^+^, 148 cells/mL) showed a remarkable increase in CD4^+^ cells to 651 and 529 cells/mL, respectively when spontaneous clearance of CHC was achieved. However, in *Case 2*, a cirrhotic patient, CD4^+^ reconstitution after effective use of cART was not observed. Along similar lines, Endo *et al*. [14] reported the case of a man whose CD4+ cell count was stable for 8 years before a change of cART regimen, so that the change in regimen was the only explanation for HCV viral resolution. Nonetheless, in the context of immune reconstitution, almost all reported cases of spontaneous clearance of CHC have linked it to the increase in CD4^+^ cell count after effective use of cART [13, 15–19].

Most HIV/HCV co-infected patients who initiate cART have immune reconstitution [21]. In our series, however, only 3 (0.59%) of 509 patients with CHC were identified with spontaneous clearance of CHC after initiating cART. It may be speculated therefore that other factors are necessary in order to achieve spontaneous clearance of CHC after effective cART. Factors involved in the immune response, such as IL28B, have been widely associated with spontaneous resolution of HCV acute infection and are also predictive of response to HCV treatment. In the context of spontaneous clearance of CHC, *Vispo* et al. [17] found that all patients who experienced spontaneous clearance of CHC (n = 6) bore the IL28B-CC genotype. Likewise, *Stenkvist* et al. [18] described 3 patients in his series who carried the favorable genotype. As was the case in those studies, all the patients in ours who experienced spontaneous CHC clearance also had the same favorable genotype. This suggests that the IL28B genotype may be an important factor for the spontaneous clearance of CHC. On the other hand, HCV genotype 3 has been associated with a higher rate of acute self-limiting HCV infection [22], so that spontaneous clearance of chronic HCV infection with genotype 3 is less frequent than in infections caused by other HCV genotypes. In our series, 2 of 3 cases (the remaining case was not available) were infected with genotype 3. In this line, *Grint* et al. [19] also described 9 cases of spontaneous clearance CHC and 5 of them (55.6%) were patients with genotype 3. This suggests that, as was observed for the spontaneous resolution of acute HCV infection, HCV genotype 3 infection could be a favorable factor for the spontaneous clearance of CHC. More studies in this area are needed.

Several limitations should be noted. Due to the retrospective design of this study, HCV RNA testing was not determined on a regular basis in all patients. One consequence of this may be that the event was underestimated. At the same time, due to the low number of cases presented in this series, statistical differences among possible associated factors could not be established.

In conclusion, the spontaneous clearance of CHC is a rare event in the context of HIV/HCV co-infected patients. These data however could have some impact on the management of HIV/HCV co-infected patients, since treatment-naïve patients, both HCV and HIV, could benefit from the possibility of spontaneous clearance of CHC when cART is administered.
